# The pH-Driven Distribution and Migration of Phosphate, Fluoride and Metals/Metalloids in Phosphogypsum Stacks: Insights from Southwest China

**DOI:** 10.3390/molecules31061052

**Published:** 2026-03-22

**Authors:** Yongliang Sun, Mei Zhang, Dapeng Luo, Quan Long, Weiguang Guo, Jiang Hou, Le Chang, Yuqi Han, Xiaoxi Peng, Yiqian Tao, Hongjin Tong, Hongbin Wang

**Affiliations:** 1 Sichuan Academy of Eco-Environmental Sciences, Chengdu 610041, China; syliang825@163.com (Y.S.); zmeijy@163.com (M.Z.); 13668139173@163.com (D.L.); longquann63@163.com (Q.L.); guoweiguang86@outlook.com (W.G.); houjiangg@163.com (J.H.); 15198266885@163.com (L.C.); 18030603923@163.com (Y.H.); pxx2624@gmail.com (X.P.); 15198057663@163.com (Y.T.); 2 School of Architecture and Civil Engineering, Chengdu University, Chengdu 610106, China; 3 College of Architecture and Environment, Sichuan University, Chengdu 610227, China

**Keywords:** phosphogypsum, covering methods, collaborative migration of pollutants, source–sink model

## Abstract

The long-term accumulation of phosphogypsum (PG) stacks has caused combined pollution of total phosphorus (TP), fluoride (F^−^), metals and metalloids (MMs), posing a severe threat to regional ecological security. To clarify the migration characteristics of pollutants in PG stacks, water leaching experiments and environmental risk assessment were conducted in 21 typical PG stacks in Southwest China. The spatial differentiation and vertical migration characteristics of pollutants under various coverage measures (high-density polyethylene (HDPE) film covering, soil covering, a composite of film–soil covering, and open-air storage) at different pH conditions were systematically analyzed. Results indicated that under open-air stockpiling conditions, the surface accumulation of TP and F^−^ was the most significant among all covering measures, corresponding to the highest environmental risk. In contrast, the membrane–soil composite covering exhibited the optimal inhibitory effect on the surface diffusion of TP and F^−^, but was less effective for metal and metalloid enrichment. Under acidic conditions (pH < 6), the vertical migration capacity of TP, F^−^, and MMs (Cu, Cd, Cr, Pb, and Zn) increased, leading to enrichment in the deep layers of the stack. With the increase in pH, the calcium-mediated precipitation–adsorption effect created a “geochemical barrier”, facilitating the solid-phase fixation of pollutants. A significant positive correlation among pollutants indicates synergistic release and fixation behaviors. In addition, a pH-controlled P-F-MM source-to-sink conceptual model was established, outlining the dissolution, precipitation, adsorption, fixation and re-enrichment pathway from fresh stock to leachate. This work provides insights for optimizing cover designs and pollution control strategies.

## 1. Introduction

Phosphogypsum (PG) is a primary industrial solid waste produced during the manufacturing of wet-process phosphoric acid [1]. Its long-term accumulation presents significant environmental challenges worldwide. Annually, nearly 300 million tons of PG is generated globally, with China contributing about 15% of this total [2]. The vast accumulation of PG exceeds 7 billion tons worldwide; however, the comprehensive utilization rate remains a mere 15% [3,4]. The environmental hazards associated with PG arise from its geochemical composition. During the production process, impurity elements in phosphate rock, such as phosphorus (P), fluorine (F), metals and metalloids (MMs), along with residual free phosphoric acid, become embedded in the crystal structure of PG or adhere to its surface [5,6]. In both active and improperly sealed landfills, these components are gradually released through precipitation leaching, creating a persistent pollution source for the surrounding soil and groundwater systems [7].

To address and manage environmental pollution from P, F, and MMs in PG stacks, researchers have studied the migration and transformation behaviors of these pollutants extensively. Zhao [8] et al. discovered that P components in PG are highly leachable, with F migration primarily being influenced by water-soluble F. The geochemical speciation of F is significantly affected by the environmental pH. Under acidic conditions, the solubility and migration of fluoride ion (F^−^) and total phosphorus increase [9]. Conversely, in alkaline environments, these ions react with calcium (Ca^2+^) to form insoluble calcium phosphate (Ca_3_(PO_4_)_2_) and calcium fluoride (CaF_2_) precipitates, limiting their migration [8,10,11]. Lieberman [12] et al. highlighted that soluble minerals in PG alter leachate composition through selective dissolution and reprecipitation. The dynamic equilibrium between mineral salt formation and dissolution significantly impacts the release and migration of pollutants. Meanwhile, Ben Garali [13] identified cadmium (Cd), nickel (Ni), and copper (Cu) as key pollutants in PG from a specific region using pollution index assessments. Column leaching experiments demonstrated that the PG matrix effectively retains trace HM like lead (Pb), zinc (Zn), and Cu, keeping their concentrations in leachate below regulatory limits [14]. Additionally, there is a co-occurrence of pollution from perfluoroalkyl and polyfluoroalkyl substances (PFASs), alongside HM [15].

Transitioning from laboratory-scale experiments to real-world stockpile management and from focusing on single pollutants to addressing combined pollution presents significant challenges. These challenges hinder the development of precise strategies for prevention, control, and resource utilization. Most research to date has concentrated on characterizing terminal leachate [16,17,18,19,20] or conducting leaching simulations at the laboratory level [14,21,22,23,24]. However, there is a notable gap in systematically characterizing the three-dimensional spatial distribution of pollutants in actual stockpiles under heterogeneous conditions. Specifically, there is no unified on-site geochemical evidence chain to elucidate how pH influences the synergy and differentiation in the migration of pollutants such as P, F, and MMs. This process involves activation–leaching mechanisms like proton attack, competitive adsorption, and surface charge transformation, as well as precipitation fixation processes like hydroxide/calcium salt precipitation and enhanced specific adsorption. Furthermore, most studies have focused on pollutant migration within individual or a limited number of stockpiles [25,26,27,28]. There is a lack of large-scale sampling studies of PG stockpiles within a specific region, which hampers the ability to comprehensively understand the general patterns and regional variations in pollutant migration. Additionally, common treatment methods for PG stockpiles include surface film covering, vegetation covering, and composite covering [29,30,31]. Although these covering technologies are considered effective engineering controls, there is insufficient systematic evaluation of their long-term effectiveness in controlling pollutant migration and assessing potential environmental risks.

This study examines several typical PG stockpiles in Southwest China, employing systematic grid sampling for the first time to gather surface and longitudinal profile samples. These samples encompass four typical scenarios: high-density polyethylene (HDPE) membrane coverage, soil coverage, membrane–soil composite coverage, and open storage. The research aims are: (1) to quantitatively characterize the spatial distribution and surface inventory of pollutants such as P, F, and MMs under different coverage methods; (2) to uncover the vertical migration patterns of pollutants and elucidate how pH, as a key environmental factor, drives the synergistic migration of P, F, and MMs; and (3) to construct a conceptual model of pollutant migration based on pH control, inferring the dynamic evolution of pollutants from source to sink. This study will provide practical strategies for accurate early-warning systems for risks and systematic management of PG stockpiles.

## 2. Materials and Methods

### 2.1. Sampling Area

**Figure 1:**
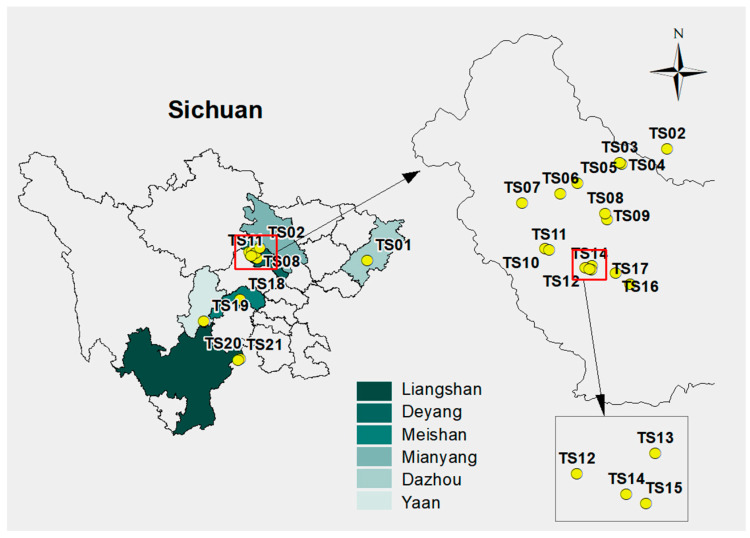
Location of the PG stack yard.

**Figure 2:**
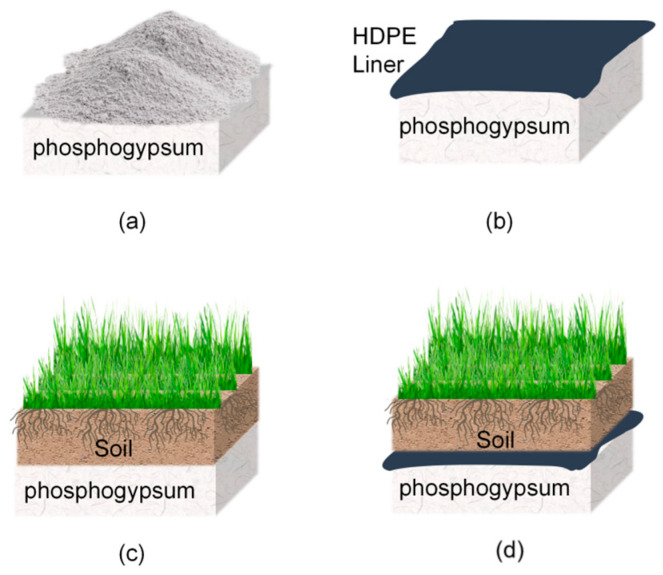
(**a**) Open-air storage; (**b**) film covering; (**c**) soil covering; (**d**) film–soil composite covering.

Figure 1 illustrates the locations of the 21 representative phosphogypsum (PG) stacks (TS01–TS21) in Southwest China according to geographical distribution, site attributes, and field accessibility. These stockpiles are situated across six cities in Sichuan Province, with Deyang City serving as the primary gathering place. These stockpiles are managed through four typical engineering treatment modes (Figure 2): HDPE film covering (Film), soil covering (Soil), a composite of film–soil covering (Film + Soil), and open-air storage (Open).

### 2.2. Sample Collection

**Figure 3:**
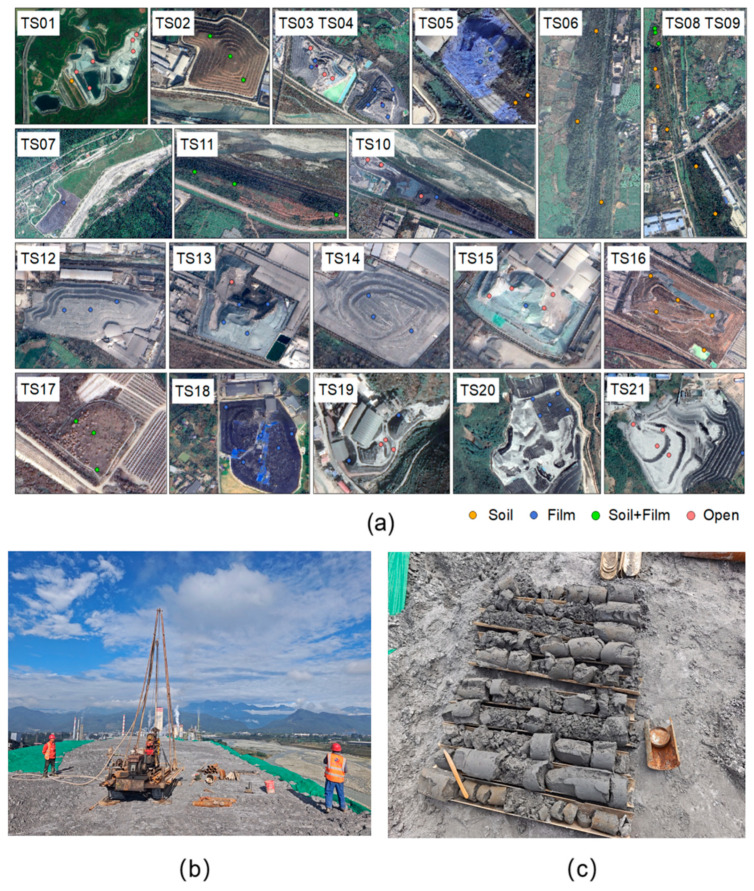
(**a**) Layout of sampling points, (**b**) vertical drilling process, (**c**) and undisturbed PG samples.

A planar grid method was applied within each stockpile, with grid shapes being tailored to the topography (Figure 3a). Three to five sampling points were randomly selected within each grid. The sampling depth was determined by the stockpile’s structure—the surface layer at the interface between the coverage and PG, the middle layer (one-third of the stockpile’s thickness), and the deep layer (two-thirds of the stockpile’s thickness)—with a maximum depth of 49 m [32]. Professional drilling equipment (Figure 3b) and dry drilling technology were employed to minimize disturbance during drilling and preserve the original physical and chemical properties of the PG samples [23]. Figure 3c presents a representative sample. A total of 223 valid samples were collected in September and October 2025. After collection, PG samples were promptly sealed and refrigerated at 4 °C to maintain stability. All samples were analyzed within one month of collection to minimize alterations during storage.

### 2.3. Water Leaching Test

The water leaching test was conducted according to SW 846 Method 1316 [33]. Initially, all PG samples were air-dried and sieved through a 2 mm nylon mesh. The moisture content was determined using the gravimetric method. For the leaching test, 20.00 g of each sample was combined with deionized water at a liquid-to-solid ratio of 10:1 (mL/g) in a high-density polyethylene bottle equipped with a leak-proof cap. These samples were then placed in a THZ-C-1 (Peiying, Suzhou, China) desktop constant-temperature oscillator, oscillating at 120 ± 2 rpm for 48 h at room temperature (20 ± 1 °C). After oscillation, the samples were filtered through a 0.45 µm membrane filter. The pH of the leachate was measured by a pH meter (PHS-3E, Leici, Shanghai, China). The concentrations of Cu, Cd, Cr, Ni, Pb, and Zn were analyzed using a NexION 2000 inductively coupled plasma mass spectrometer (ICP-MS, PerkinElmer, Waltham, MA, USA), while As and Hg concentrations were determined via an atomic fluorescence spectrometry (AFS-8220, Beijing Titan Instruments, Beijing, China). Anion concentrations, specifically F^−^ and PO_4_^3−^, were assessed using ion chromatography (ECO IC, CIC-D100, Qingdao Shenghan Chromatography Technology, Qingdao, China).

During the experiment, samples were randomly selected for repeated parallel analysis to ensure accuracy. Certified reference materials (CRMs) were employed for both spike recovery and CRM verification. In the parallel sample analysis, the relative deviations among all repeated samples adhered to the specified tolerance limits: a standard deviation for corrosive pH of ≤0.15, relative deviations for MMs of ≤10% to 35%, and for F^−^ and TP of ≤10%. No deviations exceeded these allowable ranges. In the spike recovery experiment, recovery rates for each target substance, including As, Cu, Cd, Cr, Hg, Ni, Pb, and Zn, ranged from 70.0% to 110%. The reference materials used included BY400029, NST-4, and the GSS series (such as GSS-2a, GSS-3a, GSS-5, GSS-7, GSS-34), along with BW series reference materials, all sourced from the Institute of Geophysical and Geochemical Exploration, Chinese Academy of Geological Sciences.

### 2.4. Metal and Metalloid Pollution Index

The pollution evaluation criteria were based on the risk screening values outlined in the Soil Environmental Quality–Risk Control Standard for Soil Contamination of Agricultural Land (Trial) (GB 15618-2018 [34]) and the standard limits specified in the Integrated Wastewater Discharge Standard (GB 8978-1996 [35]). To assess the pollution risk level of MMs in the PG repository, the single-factor pollution index (SFPI) and the Nemerow comprehensive pollution index (NCPI) were employed. The detailed surface metals/metalloids dataset and the corresponding pollution assessment results are provided in Table S1 of the Supplementary Materials. The calculation formulas for these indices are as follows:

(1) $$P_{i}\;={\;C}_{i}/S_{i}$$

(2) $$P_{N}=\sqrt{\frac{(P_{i,\mathrm{ave}}{)}^{2}+(P_{i,\max}{)}^{2}}{2}}$$

In the formula, *P_i_* denotes the SFPI value for metals and metalloids i, where *C_i_* represents the content of i, and *S_i_* is its standard evaluation value. *P_N_* stands for the NCPI value, while *P_ave_* refers to the large value. In this study, *P_i_* and *P_max_* are defined as the average and maximum SFPI values, respectively. *P_i_* < 1.0, and *P_i_* ≥ 1.0 indicate that the element is safe and excessive, respectively [36].

### 2.5. Statistical Analysis

The data were preprocessed using OriginLab Pro 2025 software. To identify outliers, box plots were utilized in conjunction with the 1.5 × IQR rule. Pearson correlation analysis assessed the relationships among pollutant contents, with statistical significance being determined at *p* < 0.05.

## 3. Results and Discussion

### 3.1. Spatial Differentiation of Pollutants

**Figure 4:**
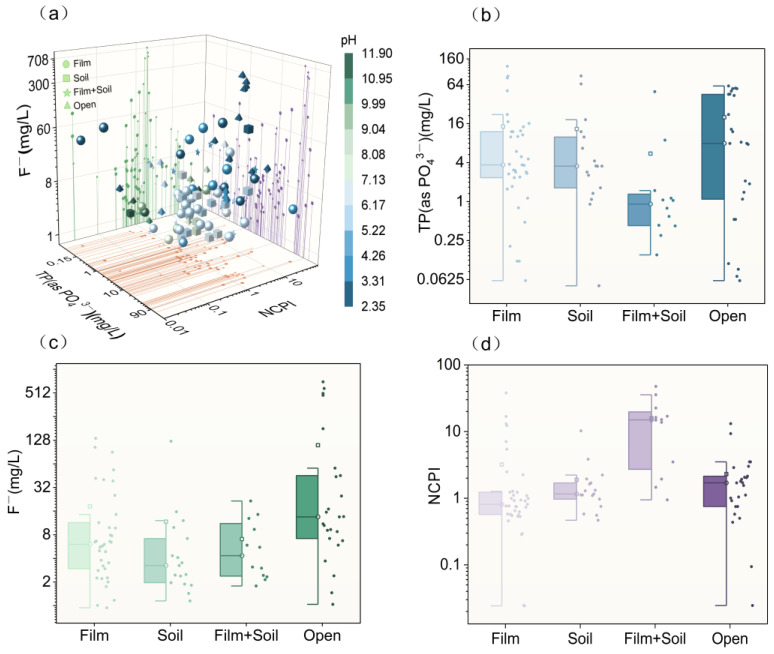
(**a**) Spatial distribution of surface PG pollutants; (**b**) TP(as PO_4_^3−^) concentration; (**c**) F^−^ concentration; (**d**) MM NCPI.

In a study examining pollutant distribution on the surface of a PG stack yard, 91 samples were collected, revealing significant heterogeneity. Figure 4a illustrates the impact of various covering methods on soil pH levels. PG covered with soil (Soil), HDPE film (Film), and a combination of both (Soil + Film) generally exhibited pH levels from slightly acidic to neutral. In contrast, the open-storage PG (Open) was more acidic. The observed pH hierarchy was Open (2–4) < Film (3.5–6) < Soil (5–7) < Soil + Film (5–7). This pattern is attributed to the properties of PG, a by-product of wet-process phosphoric acid production, typically having a pH between 1 and 4 [37]. It contains acidic components such as free phosphoric acid and trace-soluble PO_4_^3−^. Without protective barriers like soil or film, these acidic components and hydrolysis products diffuse rapidly due to atmospheric precipitation and surface runoff, significantly lowering the surface environment’s pH and causing acidity. External factors also contribute to significant pH fluctuations. When soil or film is used as a cover, they act as physical barriers, preventing the migration of acidic components from the PG [38,39,40]. This barrier also reduces water contact, inhibiting hydrolysis reactions and minimizing acidic substance infiltration, thus preventing a rapid pH drop. Soil, in particular, contains clay minerals, organic matter, and carbonates that can neutralize some acidic substances [41,42]. Additionally, soil provides a medium for vegetation growth, where microbial activities in the soil–vegetation system enhance buffering capacity by transforming acidic substances and regulating the acid–base balance, maintaining the pH at a neutral to slightly acidic level. Using both film and soil as a cover offers dual protection, sustaining a slightly acidic to neutral pH and minimizing pH fluctuations more effectively than soil alone.

The TP (as PO_4_^3−^) concentration in the surface layer varies with different covering methods, ranked as follows: Open > Film > Soil > Soil + Film (Figure 4b, Table 1). Similarly, the F^−^ concentration follows a comparable pattern: Open > Film > Soil ≈ Soil + Film (Figure 4c, Table 1). This distribution is linked to surface pH. In open stacking PG, the absence of a physical barrier allows acidic components to rapidly lower the pH through precipitation leaching. This increased acidity enhances the dissolution and migration of phosphate and F^−^, leading to higher pollutant concentrations. Conversely, when coverings are applied, the surface pH tends to be slightly acidic but closer to neutral, effectively inhibiting the diffusion and migration of phosphate and F^−^. Notably, under composite covering (Soil + Film), the pH remains more stable, resulting in lower pollutant concentrations. Single covering methods, however, experience more fluctuations due to factors such as film aging and damage, as well as the complex mechanisms of biological fixation, adsorption, and the slow release of phosphate by the soil. Despite these fluctuations, pollutant levels remain consistently lower than those in open stacking areas [43,44,45,46].

It is interesting that the survey indicated that the Nemerow Composite Pollution Index (NCPI) for MMs in the surface layer of PG generally ranks as follows: Soil + Film > Open ≈ Soil > Film (Figure 4d, Table 1). The composite covering PG (Soil + Film) exhibited the highest pollution levels (median NCPI: 14.89; mean: 15.89), significantly exceeding other covering methods. This severe pollution is primarily due to two factors. First, the composite covering effectively blocks rainwater, preventing the leaching of MMs and causing their prolonged accumulation in the PG surface layer [47]. Second, this covering creates a semi-anaerobic or anaerobic environment, which promotes the transformation of certain MMs, such as Cd^2+^, Pb^2+^, Zn^2+^, into stable and insoluble forms like hydroxides or sulfides [48,49,50].

(3) $$M^{2+}+S^{2-}\;⇌\;\mathrm{MS}(s)$$

(4) $$M^{2+}+HS^{-}\;⇌\;\mathrm{MS}(s)+H^{+}$$

Owing to the absence of morphological loss during detection, the total concentrations measured under these conditions are correspondingly higher than those observed under alternative scenarios. Under the single film covering condition, the NCPI exhibited significant fluctuations, with an average of 3.20, which was higher than the average value of the open-air condition. However, the median value was 0.81, lower than that of the open-air condition. This fluctuation may stem from varying degrees of sealing in the covering. Field investigations revealed that some membranes were damaged due to inadequate soil cover protection. These breach points act as preferential pathways for pollutant migration, where concentrated infiltration of precipitation induces focused leaching of the underlying phosphogypsum, thereby creating localized contamination hotspots with anomalously high NCPI values, as observed at several sampling points in stockpile TS13. Another possible reason is that, for most enterprises, the film covering duration is shorter than the detection period. By the time of detection, prolonged rain exposure may have reduced the metal and metalloid contents to a relatively low level. NCPI values under single soil covering and open-air conditions are essentially similar. This similarity may be due to the soil covering condition allowing for normal gas and water exchange without completely preventing rain leaching. Additionally, plant and microbial activities help reduce MM accumulation through plant absorption and microbial fixation, resulting in an overall level that is slightly lower than that in the open-air condition [51].

In summary, various covering methods influence the release and accumulation of TP (as PO_4_^3−^) and F^−^ and help inhibit MM migration by regulating the pH of the reservoir area’s surface layer. Of these methods, Soil + Film covering is the most effective for stabilizing pH and reducing surface pollutant concentrations, particularly in controlling the diffusion of TP (as PO_4_^3−^) and F^−^. Despite its effectiveness, composite covering has limitations in preventing MM migration. Therefore, additional strategies are necessary to effectively manage and control surface MMs in PG.

### 3.2. Vertical Migration of PO_4_^3−^ and F^−^

#### 3.2.1. Vertical Migration of PO_4_^3−^

**Figure 5:**
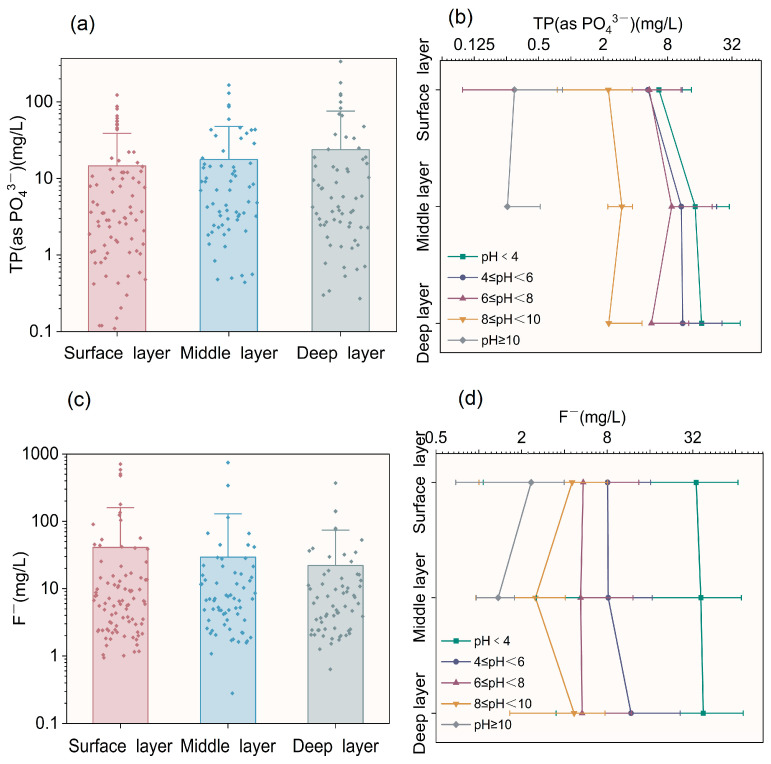
(**a**) TP (as PO_4_^3−^) concentration distribution across various depths (**b**) and longitudinal distribution of TP (as PO_4_^3−^) at varying pH levels. (**c**) Concentration distribution of F^−^ at different depths; (**d**) longitudinal distribution of F^−^ at different pH values.

In the analysis of the vertical migration of PO_4_^3−^, 223 data points were collected, with 197 being deemed valid. A total of 26 statistical outliers were excluded based on a 1.5 × IQR criterion applied to a boxplot constructed in Origin (Figure 5a), primarily found under strongly acidic conditions (pH < 4). These outliers showed relatively high phosphate concentrations, with a peak of 337 mg/L in the deep layer of PG stack. This suggests long-term acid accumulation in the PG stack, leading to continuous phosphate release. The valid sample analysis in Table 2 reveals that PG stacks in Sichuan Province are generally acidic. Comparing valid samples across pH ranges, the largest sample size was found in the 4 ≤ pH < 6 range. This indicates that this pH range is crucial for studying vertical migration of pollutants and is valuable for understanding its environmental migration and transformation mechanisms.

After removing outliers, the data show a significant negative correlation between phosphate concentration and pH (Figure 5b). Within the complex field system, the phosphate concentration showed a strong negative correlation with pH. The average concentration decreased from 16.63 mg/L to 0.255 mg/L with increasing pH, indicating that the pH significantly influences its migration. Under acidic conditions (pH < 6), the TP (as PO_4_^3−^) concentration rises with depth. Meanwhile, 6 ≤ pH < 8, TP (as PO_4_^3−^) continues to migrate downward but is mostly confined to the surface and middle layers. In alkaline conditions (8 ≤ pH < 10), the TP (as PO_4_^3−^) distribution becomes uniform, and the overall concentration remains low, with an average maximum of 2.99 mg/L. When pH ≥ 10, the concentration dropped to its lowest level at 0.255 mg/L, and no deep-layer samples were found in this strongly alkaline range.

The differences in phosphate dissolution and fixation mechanisms under varying pH conditions primarily account for these observations. In acidic environments, high concentrations of H^+^ facilitate the dissolution of insoluble phosphorus-containing minerals in PG. These ions also protonate adsorption sites on particle surfaces, causing the desorption of PO_4_^3−^ fixed through inner-sphere complexation [52], thereby increasing its solubility and vertical migration capability. This aligns with Dong [53] et al.’s findings, which reported a significantly higher leaching of PO_4_^3^^−^ under acidic conditions. While OH^−^ may induce some phosphate desorption through competitive adsorption under alkaline conditions, the concurrent and dominant precipitation of phosphate with Ca^2+^ (forming minerals such as Ca_3_(PO_4_)_2_ and Ca_5_(PO_4_)_3_OH) and other metal ions [54,55,56,57] results in its effective fixation into the solid phase and a pronounced decrease in leaching concentration.

#### 3.2.2. Vertical Migration of F^−^

A total of 210 valid data points were collected to analyze the vertical migration of F, with 13 outliers being identified and excluded (Figure 5c, Table 3). These outliers were found under strongly acidic conditions, where the average pH < 2.53 resulted in extremely high F^−^ concentrations. The peak concentration at the middle layer reached 746 mg/L. In the deeper layer, the extension of the seepage path, along with the potential formation of CaF_2_ precipitation or adsorption by clay minerals, weakened the accumulation of strong acidity. Consequently, high-concentration anomalies were less likely to form.

After excluding outliers, there was a significant coupling relationship between F^−^ concentration and pH (Figure 5d). Under neutral to acidic conditions (pH < 8), the overall level of F^−^ was relatively high. The maximum average concentration in a strongly acidic environment reached 35.63 mg/L, and the vertical distribution was relatively uniform. When pH ≥ 8, the F release decreased significantly, and the migration ability weakened. The concentration in the middle layer (2.36 mg/L) was lower than that in the surface layer (4.57 mg/L). Due to long-term stacking, F^−^ was enriched in the deep layer, and its concentration exceeded that in the middle layer.

The distribution of F is primarily influenced by its speciation transformation and the solid–liquid partitioning mechanism. In acidic conditions, fluorine (introduced from sources such as NaF or KF) is transformed into fluorosilicate (SiF_6_^2−^) and predominantly exists in the form of HF and H_2_SiF_6_ [58], which are susceptible to vertical migration through seepage. In strongly acidic environments (pH < 4), the saturation indices of CaF_2_ and rare-earth fluorides (YF_3_) are low. This indicates that these minerals have not reached dissolution–precipitation equilibrium, resulting in high solubility and migration activity of F. Conversely, in alkaline conditions, the saturation indices of CaF_2_ and YF_3_ increase significantly, promoting precipitation. Experiments demonstrate that in systems with high pH and high Ca^2+^ concentrations, CaF_2_ can precipitate stably, thereby significantly reducing the F concentration in the aqueous phase [11,59,60]. Additionally, F^−^ can be adsorbed onto the surfaces of Fe/Al oxides or minerals through adsorption and site-competition mechanisms [61]. The combination of these effects inhibits vertical migration of F in alkaline environments, leading to only localized enrichment in deeper layers due to prolonged accumulation.

In summary, in PG stacks, if the stacking or underlying medium is rich in Ca and/or Fe/Al minerals, a highly alkaline environment can facilitate the coupling of CaF_2_ precipitation and surface adsorption on minerals, effectively limiting the deep migration of F^−^. Additionally, the vertical movement of F in PG stockpiles may be influenced by other, less understood processes. These include the slow transformation of surface-bound fluoride, regulation of F speciation by organic matter or microbial activities, and microscopic variations in water–rock interactions within heterogeneous pore structures. These potential mechanisms, along with their interactions with primary controlling factors such as pH, coexisting ions, and mineral composition, require further investigation. Future studies should focus on microscopic characterization, dynamic simulation, and long-term monitoring to elucidate these effects.

### 3.3. Collaborative Migration of Multiple Pollutants

**Figure 6:**
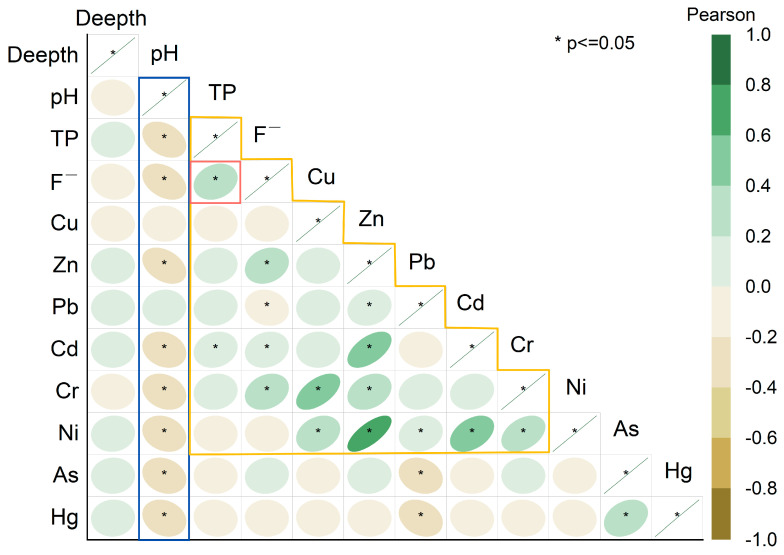
Correlation analysis of pollutants in PG stored in the warehouse.

Figure 6 reveals a general negative correlation between these pollutants and pH levels, indicating the pivotal role of pH in regulating pollutant migration. Additionally, TP and F^−^ concentrations show a strong positive correlation, which is particularly pronounced under low-pH conditions. This suggests that these pollutants tend to migrate synergistically, with simultaneous release and precipitation.

From a geochemical perspective, acidic conditions increase the solubility of minerals such as Ca_3_(PO_4_)_2_ and CaF_2_, thereby mobilizing phosphate and F^−^ into the aqueous phase and facilitating their vertical migration with percolating water. In contrast, in neutral to alkaline environments, phosphate and F^−^ readily combine with Ca^2+^ to form insoluble precipitates like Ca_5_(PO_4_)_3_OH and CaF_2_. This reaction limits their concentrations in the aqueous phase and restricts their migration. Research has shown that when Ca^2+^ and phosphate coexist in the system, F^−^ can be incorporated into the apatite lattice through co-precipitation, forming fluorapatite (Ca_5_(PO_4_)_3_F), while phosphate is stabilized as calcium phosphate. This process effectively passivates both ions [62,63,64,65]. In conclusion, this study demonstrates that the pH gradient and Ca^2+^ concentration play pivotal roles in, and are likely among the primary controls of, the synergistic migration of TP and F^−^ in the PG stockpiles.

Figure 6 illustrates a generally positive correlation among MMs like Cu, Cd, Cr, Pb, and Zn. This suggests a clustering effect characterized by co-distribution, co-enrichment, and co-migration of these metals within the PG stack. Supporting this, Dai [66] et al. used field sampling and geostatistical analysis to demonstrate that the spatial distributions of Cr, Cu, Cd, Pb, and Zn are significantly positively correlated, suggesting a common source or shared mechanisms of migration, deposition, and enrichment. Similarly, Feng’s [67] research confirmed that the spatial correlation between Pb and Ni is influenced by both natural soil-forming processes and anthropogenic pollution inputs.

TP and F^−^ show a strong positive correlation with Zn, Cd, and Cr, indicating a coupled migration relationship between F, P, and these MMs. This relationship is primarily driven by co-precipitation. Studies demonstrate that under high-pH conditions, F^−^ and MMs can co-precipitate synergistically with Ca^2+^ and phosphate, forming complex insoluble minerals [68]. This process effectively reduces the concentrations of various pollutants in the aqueous phase. This suggests that MM migration is not an isolated process but part of a multi-component “co-migration” mode, involving precipitation, adsorption, and mineralization.

This study integrates observational data with existing research to propose a hypothesis on the synergistic migration of multiple pollutants in PG stockpiles. It suggests a significant coupling effect in the migration of TP, F^−^ and MM, primarily influenced by environmental pH. In acidic conditions, a low pH leads to acid erosion, breaking down mineral lattices containing P, F, and MMs. This process simultaneously releases these pollutants into the aqueous phase, creating an “activated state” of synergistic migration. Conversely, in neutral to alkaline environments, Ca^2+^ facilitates the formation of Ca_3_(PO_4_)_2_ and CaF_2_ or Ca_5_(PO_4_)_3_F precipitates. Concurrently, MM ions are immobilized through co-precipitation or adsorption. This results in a “passivated state,” significantly reducing the pollutants’ downward migration. Notably, in high-calcium systems, phosphate-F- Ca and MM composite precipitates form a natural “geochemical barrier” within the stockpile, effectively slowing the deep migration of pollutants. This hypothesis aligns with classic thermodynamic findings on Ca_3_(PO_4_)_2_ and calcium F^−^ precipitates and is strongly supported by the observational data presented in this study [62,63,69,70].

In summary, the synergistic migration mechanism of P-F-MM in the PG stockpile is significantly influenced by pH levels. This migration behavior is regulated by various geochemical processes, including dissolution–precipitation and adsorption–desorption, and generally exhibits a pattern of “acid activation and alkali/neutral passivation.” Future research should aim to validate this synergistic migration hypothesis through column leaching tests, percolation simulation experiments, and multi-pollutant dissolution–precipitation thermodynamic tests. Additionally, it is essential to quantify the migration rates and conversion efficiencies of pollutants under varying pH and Ca^2+^ concentration conditions. These efforts will provide more precise theoretical support for the prevention, control, and remediation of pollution in PG stockpiles.

### 3.4. Conceptual Model of the Entire Source-to-Sink Migration Process

**Figure 7:**
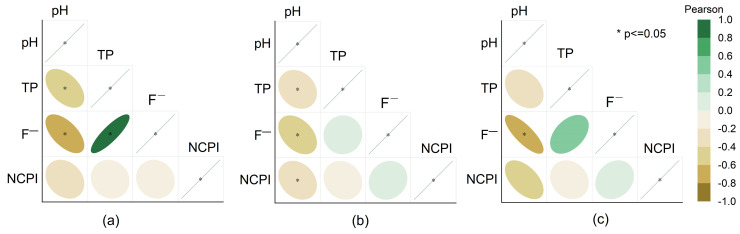
Correlations of pollutants in fresh PG (**a**); correlations of pollutants in PG stored in the stockpile (**b**); correlations of pollutants in leachate (**c**).

In the fresh phase, the correlation heatmap (Figure 7a) shows a positive correlation between TP and F^−^ that could be associated with source minerals and release synchronously. Table 4 shows that the pH level of fresh PG is 4.72. In this acidic environment, rainfall leaching facilitates entry of P and F into the solution by mineral dissolution. The negative correlation between pH and these pollutants shows that acidic conditions enhance release. At this stage, the main mechanism for the migration of pollutant is “mineral dissolution–liquid phase leaching”.

After entering the reservoir’s storage stage (Figure 7b), TP remains positively correlated with F^−^, but the correlation strength declines markedly, indicating that the reservoir’s complex physical and chemical processes alter their migration rates and progressively restrict their free transport.

Correlation analysis of pollutants (Figure 6) indicates that changes in reservoir pH favor water–solid interface reactions as the dominant migration pathway. In particular, phosphate and F^−^ can react with Ca^2+^ to form insoluble precipitates such as Ca_3_(PO_4_)_2_ and CaF_2_ or Ca_5_(PO_4_)_3_F, resulting in solid-phase fixation of these contaminants. Concurrently, the strong positive correlations among MMs (Cu, Zn, Pb, Cd), and their notable correlations with PO_4_^3^^−^ and F^−^, imply that MMs undergo similar migration mechanisms—co-precipitation, adsorption, and mineralization—leading to a “cluster retention” effect. Together, these processes enhance pollutant retention in the reservoir and reduce their potential for vertical migration.

In the leachate stage (Figure 7c), positive correlations among pollutants like TP, F^−^, and MMs suggest their synergistic migration into the leachate system, characterized by high concentration and enrichment. According to Table 4, the average concentration of TP (as PO_4_^3^^−^) in the leachate was 819.71 mg/L, peaking at 4630 mg/L. The average concentration of F^−^ was 317.21 mg/L, with an NCPI value of 7.60, both significantly exceeding levels in fresh PG and during the pond storage stage. This indicates that during stockpiling, toxic substances are released from the PG matrix into the leachate, significantly enhancing their mobility and pollution diffusion potential. Consequently, it is crucial to strengthen terminal treatment and risk management of the leachate [16,71,72,73].

**Figure 8:**
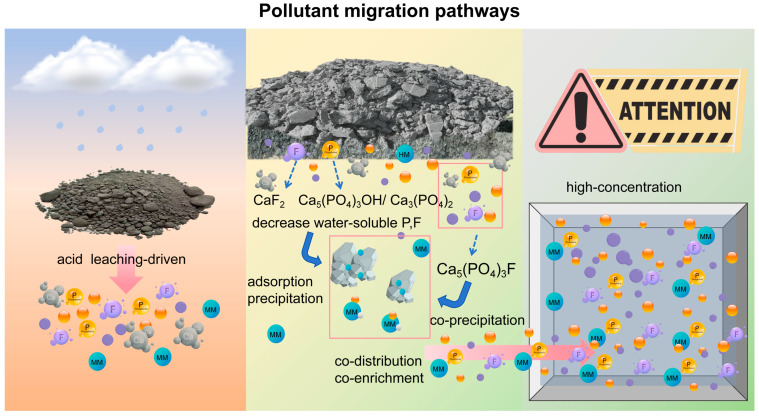
A flow chart illustrating the entire migration process of TP, F^−^, and MMs in PG, tracing their journey from the source to the leachate.

Figure 8 illustrates the migration process of pollutants from fresh PG to the repository stockpile and eventually to the leachate. This process involves a gradual transformation through stages of “dissolution–precipitation–adsorption–fixation–re-enrichment.” Initially, at the source, P and F are released simultaneously due to the acidic environment. In the repository, pollutants are retained in the solid phase through pH-mediated interfacial reactions. In the leachate stage, non-immobilized pollutants are synergistically enriched and diffused. This sequence not only highlights the evolution of pollutants’ physical and chemical forms but also underscores the critical role of pH-driven dissolution, precipitation, and adsorption in the migration of P, F, and MMs. It provides essential insights into understanding the dynamic fate and environmental risks associated with pollutants in PG stockpiles.

## 4. Conclusions

Research indicates that the migration behavior of pollutants, such as P, F, and MMs, in the PG stack is influenced by pH levels. Initially, a low-pH environment facilitates the dissolution and release of P and F. Conversely, under neutral to alkaline conditions, these pollutants become immobilized through co-precipitation and adsorption, which reduces their ability to migrate. This demonstrates the significant role of pH in controlling pollutant migration. In addition, the composite covering method was more effective than the HDPE film covering, soil covering, and open-air stockpiling method in minimizing pollutant migration, as it significantly reduced both the release and infiltration potential of pollutants, showcasing strong environmental control. Nonetheless, its effectiveness in controlling surface MM was comparatively limited.

The progression of pollutants in the PG yard from “source” to “sink” can be categorized into three distinct stages. Initially, in the fresh PG stage, “mineral dissolution–liquid phase leaching” is predominant, leading to the simultaneous release of TP and F^−^ due to their shared origin. During the in-yard stacking stage, pollutants are retained in the solid phase through precipitation and adsorption, facilitated by pH-mediated water–solid interface reactions. In the leachate stage, pollutants that have not been fixed undergo synergistic enrichment, creating a high-concentration pollution sink. Consequently, a pH-driven P-F-MM source–sink conceptual model was developed. This model comprehensively elucidates the entire process of pollutant fate, from release and retention to re-enrichment, offering a theoretical framework for phased control of yard pollution.

This research identified a characteristic synergistic migration among P, F, and MMs. Notably, under varying pH conditions, these pollutants interact during migration, resulting in co-release, co-precipitation, and adsorption fixation. This suggests that pollutant migration is not an isolated process but involves interactions through precipitation, adsorption, and co-precipitation mechanisms, forming a “co-migration” pattern.

Based on the findings of this study, future research should focus on elucidating the molecular-scale mechanisms underlying the pH-driven co-migration of P, F, metals and metalloids through advanced micro-spectroscopic techniques, while employing multivariate statistical approaches such as PCA and cluster analysis to quantitatively differentiate their sources and associations across varying depths and stockpile ages. Additionally, integrated remediation strategies combining physical covering with chemical stabilization or phytoremediation warrant investigation to address the limited efficacy of surface MM control, and long-term field monitoring under dynamic environmental conditions is essential to validate the durability of pollution control measures. Comparative studies across multiple phosphogypsum stockpiles with different histories and geographical settings would further establish a generalized understanding of pollutant migration patterns and inform site-specific remediation protocols.

## Figures and Tables

**Table 1:** Statistics of surface samples.

Coverage	Surface PG Sample Quantity	Sample Percentage	TP (as PO_4_^3−^)		F^−^		NCPI	
			Median	Mean	Median	Mean	Median	Mean
Film	37	40.66%	3.69	14.42	6.04	18.35	0.81	3.20
Soil	17	18.68%	3.53	13.24	3.24	11.74	1.12	1.88
Film + Soil	12	13.19%	0.915	5.52	4.32	7.04	14.98	15.89
Open	25	27.47%	7.92	20.05	13.5	110.32	1.70	2.30
Grand total	91	100.00%	/	/	/	/	/	/

**Table 2:** Statistical data of PO_4_^3−^.

Depth		Surface Layer	Middle Layer	Deep Layer	Total
Total sample size		91	66	66	223
Number of outlier samples	Quantity	15	5	6	26
Effective sample size	pH < 4	14	10	11	35
	4 ≤ pH < 6	34	35	32	101
	6 ≤ pH < 8	14	12	13	39
	8 ≤ pH < 10	8	2	4	14
	pH ≥ 10	6	2	0	8
	Total	76	61	60	197

**Table 3:** Statistical data of F^−^.

Depth		Surface Layer	Middle Layer	Deep Layer	Total
Total sample size		91	66	66	223
Number of outlier samples	Quantity	5	2	6	13
Effective sample size	pH < 4	19	13	7	39
	4 ≤ pH < 6	39	35	36	110
	6 ≤ pH < 8	14	12	13	39
	8 ≤ pH < 10	8	2	4	14
	pH ≥ 10	6	2	0	8
	Total	86	64	60	210

**Table 4:** Whole-process analysis.

Pollutants		Total Number of Samples	Mean	Minimum	Median	Maximum Value
Fresh PG	pH	19	4.72	2.20	3.90	7.70
	TP	19	165.00	9.73	53.10	1180.00
	F^−^	19	60.37	0.00	14.80	285.00
	NCPI	19	5.65	0.00	0.02	89.09
PG stored in the warehouse	pH	110	5.11	2.25	4.90	11.90
	TP	110	24.13	0.06	9.18	178.00
	F^−^	110	15.58	0.28	7.74	142.00
	NCPI	110	7.50	0.00	0.02	63.99
Leachate	pH	9	3.21	1.80	2.60	6.30
	TP	9	819.71	11.70	239.00	4630.00
	F^−^	9	317.21	24.30	403.00	722.00
	NCPI	9	7.60	0.17	3.67	19.83

## Data Availability

The original contributions presented in this study are included in the article/Supplementary Material. Further inquiries can be directed to the corresponding author(s).

## Supplementary Materials

The following supporting information can be downloaded at: https://www.mdpi.com/article/10.3390/molecules31061052/s1, Table S1: Surface Metals/Metalloids Dataset and Pollution Assessment (Nemerow Index) of a Phosphogypsum Stockpile. Table S2: Raw Dataset for Vertical Distribution of Contaminants. Table S3: Raw Dataset of Contaminants in Fresh PG. Table S4: Raw Data on Pollutant Constituents in PG-L. Table S5: Data Quality Control (QC).
