# Supplementary material for: The pH-Driven Distribution and Migration of Phosphate, Fluoride and Metals/Metalloids in Phosphogypsum Stacks: Insights from Southwest China
Source: Molecules. 2026 Mar 22;31(6):1052. doi: 10.3390/molecules31061052 (PMC13029643; doi:10.3390/molecules31061052)
Supplement: Supplementary file 1 [file molecules-31-01052-s001.zip › molecules-4179314-supplementary.pdf]

Supplementary Information

pH-Driven Distribution and Migration of Phosphate , Fluorideand Metals/Metalloids in Phosphogypsum Stacks: Insights from Southwest China.

Yongliang Sun [a], Mei Zhang [a, b], DaPeng Luo [a, c], Quan Long [a], WeiGuang Guo [a], Jiang Hou [a] , Le Chang [a], YuQi Han [a], XiaoXi Peng [a], YiQian Tao [a], HongJin Tong [a*] and Hongbin Wang [b*]

[a] *Sichuan Academy of Eco-Environmental Sciences, Chengdu 610041, China.*

[b] *School of Architecture and Civil Engineering, Chengdu University, Chengdu 610106, China*

[c] *College of Architecture and Environment, Sichuan University, Chengdu 610227, China.*

* *Corresponding author's E-mail: tonghongjin@scaes.cn, wanghongbin@cdu.edu.cn*

Table of contents

Table S1	Surface Metals/Metalloids Dataset and Pollution Assessment (Nemerow Index) of a Phosphogypsum Stockpile
Table S2	Raw Dataset for Vertical Distribution of Contaminants
Table S3	Raw Dataset of Contaminants in Fresh PG
Table S4	Raw Data on Pollutant Constituents in PG-L
Table S5	Data Quality Control (QC)

Table S1 Surface Metals/Metalloids Dataset and Pollution Assessment (Nemerow Index) of a Phosphogypsum Stockpile

PG Yard	Location	Coverage	pH	NCPI	Hg	As	Cu	Ni	Zn	Pb	Cd	Cr	Pi_Hg	Pi_As	Pi_Cu	Pi_Ni	Pi_Zn	Pi_Pb	Pi_Cd	Pi_Cr
TS01	TS011	Open	3.23	1.79	1.25	28.5	8.2	0	18.5	1.8	0	0	2.50	0.71	0.16	0.00	0.09	0.03	0.00	0.00
	TS012	Open	2.52	1.92	1.34	30.6	0	0	9.4	1.4	0	0	2.68	0.77	0.00	0.00	0.05	0.02	0.00	0.00
	TS013	Open	2.55	1.71	1.19	23.4	9.6	0	15.4	2.9	0	0	2.38	0.59	0.19	0.00	0.08	0.04	0.00	0.00
	TS014	Open	2.67	1.55	1.08	26.2	6.7	0	22.3	2.3	0	0	2.16	0.66	0.13	0.00	0.11	0.03	0.00	0.00
	TS015	Open	2.52	2.06	1.44	16.1	9.3	0	16.8	1.6	0	0	2.88	0.40	0.19	0.00	0.08	0.02	0.00	0.00
	TS016	Open	2.5	1.11	0.77	24.2	10.2	0	21.1	0.8	0	0	1.54	0.61	0.20	0.00	0.11	0.01	0.00	0.00
	TS017	Open	2.38	2.04	1.42	32.1	9.8	0	16.8	1.5	0	0	2.84	0.80	0.20	0.00	0.08	0.02	0.00	0.00
	TS018	Soil	2.98	2.23	1.56	12.9	7.6	0	19.4	0.6	0	0	3.12	0.32	0.15	0.00	0.10	0.01	0.00	0.00
TS02	TS021	Soil + Film	5.45	16.26	11.40	8.32	20.6	11	52.2	21.8	0	0	22.80	0.21	0.41	0.18	0.26	0.31	0.00	0.00
	TS022	Soil + Film	3.88	15.13	10.60	9.03	28.2	21.1	93.9	24.2	0	0	21.20	0.23	0.56	0.35	0.47	0.35	0.00	0.00
	TS023	Soil + Film	4.44	14.84	10.40	5.04	14.6	13.1	47.8	29	0	0	20.80	0.13	0.29	0.22	0.24	0.41	0.00	0.00
TS03	TS031	Film	5.3	2.48	1.71	5.52	24.1	31.2	39.4	49.9	0.3	0	3.42	0.14	0.48	0.52	0.20	0.71	1.00	0.00
	TS032	Open	4.1	1.00	0.29	5.11	24.1	33.5	17.4	49.9	0.4	0	0.58	0.13	0.48	0.56	0.09	0.71	1.33	0.00
	TS033	Film	6.2	0.58	0.07	4.26	23.7	33	19.6	51.4	0.3	0	0.15	0.11	0.47	0.47	0.10	0.57	0.75	0.00
	TS034	Open	5.8	0.92	0.06	4.62	15.4	37.2	30.4	29.4	0.5	0	0.12	0.12	0.31	0.53	0.15	0.33	1.25	0.00
	TS035	Open	7.3	0.44	0.24	4.62	24.8	35.2	25	66.6	0.2	0	0.39	0.15	0.25	0.35	0.10	0.56	0.33	0.00
TS04	TS041	Film	3.9	0.55	0.09	2.71	15.2	33	95.8	47.8	0.2	0	0.18	0.07	0.30	0.55	0.48	0.68	0.67	0.00
	TS042	Film	5.8	1.27	0.05	2.88	16.2	31.9	40.4	40.6	0.7	0	0.11	0.07	0.32	0.46	0.20	0.45	1.75	0.00
	TS043	Film	5.7	1.11	0.07	6.71	26.1	32.2	77.3	42.5	0.6	0	0.14	0.17	0.52	0.46	0.39	0.47	1.50	0.00
	TS044	Film	6.1	0.92	0.17	6.82	17.4	30.4	26.2	33.5	0.5	0	0.35	0.17	0.35	0.43	0.13	0.37	1.25	0.00
	TS045	Film	11	0.65	0.22	4.3	26.7	30.2	71.4	44	0.7	0	0.22	0.17	0.27	0.16	0.24	0.26	0.88	0.00
TS05	TS051	Film	5.32	0.57	0.39	9.42	10.6	1.2	9	18.2	0	0	0.78	0.24	0.21	0.02	0.05	0.26	0.00	0.00
	TS052	Film	6.26	12.30	8.62	7.77	16.3	2.6	17.8	23.2	0	0	17.24	0.19	0.33	0.04	0.09	0.26	0.00	0.00
	TS053	Film	6	5.43	3.80	5.85	12.9	0.9	10.9	29.8	0	0	7.60	0.15	0.26	0.01	0.05	0.33	0.00	0.00

PG Yard	Location	Coverage	pH	NCPI	Hg	As	Cu	Ni	Zn	Pb	Cd	Cr	Pi_Hg	Pi_As	Pi_Cu	Pi_Ni	Pi_Zn	Pi_Pb	Pi_Cd	Pi_Cr
	TS054	Soil	5.92	3.84	2.69	6.8	15.9	3.2	8.5	15.7	0	0	5.38	0.17	0.32	0.05	0.04	0.17	0.00	0.00
	TS055	Soil	5.8	10.27	7.20	4.09	4.3	0.5	10.6	21.2	0	0	14.40	0.10	0.09	0.01	0.05	0.24	0.00	0.00
TS06	TS061	Soil	11.5	0.47	0.18	4.67	24	33.3	33.3	35.2	0.5	0	0.18	0.19	0.24	0.18	0.11	0.21	0.63	0.00
	TS062	Soil	6.6	0.97	0.05	4.24	16	29.6	38.2	42.8	0.8	0	0.08	0.14	0.16	0.30	0.15	0.36	1.33	0.00
	TS063	Soil	5.9	0.75	0.11	2.05	23.9	37.4	20.6	50.4	0.4	0	0.23	0.05	0.48	0.53	0.10	0.56	1.00	0.00
TS07	TS071	Film	5.8	0.76	0.38	7.36	11.6	24.9	20.8	50.8	0.4	0	0.76	0.18	0.23	0.36	0.10	0.56	1.00	0.00
TS08	TS081	Soil + Film	5.72	35.66	25.00	6.09	7.3	5.3	34.4	25.5	0.6	0	50.00	0.15	0.15	0.08	0.17	0.28	1.50	0.00
	TS082	Soil + Film	5.59	16.99	11.90	6.2	7.8	5	41.4	31.6	0.6	0	23.80	0.16	0.16	0.07	0.21	0.35	1.50	0.00
	TS083	Soil + Film	4.42	47.64	33.40	5.11	5.8	6.2	54.7	26.4	0.6	0	66.80	0.13	0.12	0.10	0.27	0.38	2.00	0.00
TS09	TS091	Soil	6.3	1.63	0.07	4.6	18.9	39.8	34.4	54.7	0.9	0	0.14	0.12	0.38	0.57	0.17	0.61	2.25	0.00
	TS092	Soil	3.1	1.69	0.07	5.02	18.9	39.9	44.1	31.3	0.7	0	0.13	0.13	0.38	0.67	0.22	0.45	2.33	0.00
	TS093	Soil	6.4	1.10	0.05	4.17	19.7	31.4	38.3	42.6	0.6	0	0.10	0.10	0.39	0.45	0.19	0.47	1.50	0.00
	TS094	Soil	8.9	1.16	1.60	4.75	11.5	30.8	23.1	54.6	0.2	0	1.60	0.19	0.12	0.16	0.08	0.32	0.25	0.00
	TS095	Soil	8.3	1.32	1.79	3.23	12.5	30.9	58.2	14.2	1.3	0	1.79	0.13	0.13	0.16	0.19	0.08	1.63	0.00
	TS096	Soil	5.5	0.55	0.28	3.26	11.5	24.7	24.7	49.2	0.2	0	0.56	0.08	0.23	0.41	0.12	0.70	0.67	0.00
TS10	TS101	Open	3.26	9.30	6.52	33	0.21	0.11	0.229	0.16	0.01	0	13.04	0.83	0.00	0.00	0.00	0.00	0.03	0.00
	TS102	Open	4.81	0.09	0.00	5.22	0.47	0.12	0.087	0.08	0.02	0	0.00	0.13	0.01	0.00	0.00	0.00	0.07	0.00
	TS103	Open	2.84	0.02	0.00	0.91	0.71	0.29	0.348	0.11	0.01	0	0.00	0.02	0.01	0.00	0.00	0.00	0.03	0.00
	TS104	Film	2.6	0.02	0.00	0.49	1.03	0.74	0.878	0.16	0.01	0	0.00	0.01	0.02	0.01	0.00	0.00	0.03	0.00
	TS105	Film	3.14	0.02	0.00	0.65	0.43	0.2	0.314	0.09	0.01	0	0.00	0.02	0.01	0.00	0.00	0.00	0.03	0.00
TS11	TS111	Soil + Film	5.5	1.46	0.04	5.26	27.4	31	39.2	38.2	0.6	0	0.07	0.13	0.55	0.52	0.20	0.55	2.00	0.00
	TS112	Soil + Film	4.9	1.93	0.17	5.77	18.9	31.7	40.5	40.3	0.8	0	0.33	0.14	0.38	0.53	0.20	0.58	2.67	0.00
	TS113	Soil + Film	5.9	0.94	0.10	7.06	22.2	28.4	125	57	0.5	0	0.20	0.18	0.44	0.41	0.63	0.63	1.25	0.00
TS12	TS121	Film	3.4	0.75	0.11	5.3	12.5	24.7	24.3	52	0.3	0	0.22	0.13	0.25	0.41	0.12	0.74	1.00	0.00
	TS122	Film	8	0.75	0.91	6.5	12.4	22.6	22	40.9	0.8	0	0.91	0.26	0.12	0.12	0.07	0.24	1.00	0.00

PG Yard	Location	Coverage	pH	NCPI	Hg	As	Cu	Ni	Zn	Pb	Cd	Cr	Pi_Hg	Pi_As	Pi_Cu	Pi_Ni	Pi_Zn	Pi_Pb	Pi_Cd	Pi_Cr
	TS123	Film	3.1	1.22	0.82	8.13	11.4	30.5	27.6	45.9	0.3	0	1.64	0.20	0.23	0.51	0.14	0.66	1.00	0.00
TS13	TS131	Film	2.9	16.83	11.80	1.64	25.5	5.7	21.2	40.1	0	0	23.60	0.04	0.51	0.10	0.11	0.57	0.00	0.00
	TS132	Film	3.32	37.93	26.60	6.03	13.7	9	34.9	46.9	0	0	53.20	0.15	0.27	0.15	0.17	0.67	0.00	0.00
	TS133	Film	2.67	13.04	9.14	1.91	9.4	2.5	20.8	29.7	0	0	18.28	0.05	0.19	0.04	0.10	0.42	0.00	0.00
	TS134	Film	2.47	7.08	4.96	2.01	10.1	2.7	27.8	32.1	0	0	9.92	0.05	0.20	0.05	0.14	0.46	0.00	0.00
	TS135	Open	2.88	13.13	9.20	9.39	8.6	4.2	26.4	45.5	0	0	18.40	0.23	0.17	0.07	0.13	0.65	0.00	0.00
TS14	TS141	Film	5.9	0.46	0.05	6.04	21.8	31	93.4	49.9	0.2	0	0.10	0.15	0.44	0.44	0.47	0.55	0.50	0.00
	TS142	Film	5.7	0.76	0.18	7.52	26.5	30	69	38.7	0.4	0	0.37	0.19	0.53	0.43	0.35	0.43	1.00	0.00
	TS143	Film	5.4	1.23	0.08	7.81	27.6	30.2	77.8	39.2	0.5	0	0.17	0.20	0.55	0.50	0.39	0.56	1.67	0.00
TS15	TS151	Open	9.7	2.14	2.97	2.7	124	0	15.2	0	0	0	2.97	0.11	1.24	0.00	0.05	0.00	0.00	0.00
	TS152	Open	11.9	3.01	4.19	2.89	158	0	17.6	0	0	0	4.19	0.12	1.58	0.00	0.06	0.00	0.00	0.00
	TS153	Film	9.54	2.25	3.14	3	80.3	0	12.2	0	0	0	3.14	0.12	0.80	0.00	0.04	0.00	0.00	0.00
	TS154	Open	9.01	3.52	4.93	3.75	0	0	49.6	0	0	0	4.93	0.15	0.00	0.00	0.17	0.00	0.00	0.00
	TS155	Open	6.53	3.51	2.94	3.58	98.7	0	21.7	0	0	0	4.90	0.12	0.99	0.00	0.09	0.00	0.00	0.00
TS16	TS161	Soil	6.5	1.12	0.17	6.31	23.6	30.8	118	55.8	0.6	0	0.35	0.16	0.47	0.44	0.59	0.62	1.50	0.00
	TS162	Soil	5.8	1.11	0.07	4.89	23.5	35	112	52.3	0.6	0	0.14	0.12	0.47	0.50	0.56	0.58	1.50	0.00
	TS163	Soil	6.3	0.58	0.04	5.17	18.9	31.8	89.7	35.8	0.3	0	0.07	0.13	0.38	0.45	0.45	0.40	0.75	0.00
	TS164	Soil	6.4	1.29	0.11	6.74	25.2	30.5	82.4	57.4	0.7	0	0.22	0.17	0.50	0.44	0.41	0.64	1.75	0.00
	TS165	Soil	5.4	1.94	0.11	7.5	24.6	33.1	100	52.6	0.8	0	0.22	0.19	0.49	0.55	0.50	0.75	2.67	0.00
TS17	TS171	Soil + Film	3.88	22.58	15.80	6.8	55.7	59.3	56.8	24.6	0.6	0	31.60	0.17	1.11	0.99	0.28	0.35	2.00	0.00
	TS172	Soil + Film	6.13	13.76	9.63	6	13.6	11.7	83.4	21.2	0.6	0	19.26	0.15	0.27	0.17	0.42	0.24	1.50	0.00
	TS173	Soil + Film	6.4	3.50	2.43	8.13	12.5	11	53.5	28.1	0.6	0	4.86	0.20	0.25	0.16	0.27	0.31	1.50	0.00
TS18	TS181	Film	5.89	0.63	0.08	0.486	5.1	0	24.6	78.4	0	0	0.15	0.01	0.10	0.00	0.12	0.87	0.00	0.00
	TS182	Film	5.43	0.53	0.36	3.21	17.1	3.2	38.5	39.6	0	0	0.71	0.08	0.34	0.05	0.19	0.57	0.00	0.00
	TS183	Film	5.81	0.94	0.65	0.501	1.9	0	32.4	38	0	0	1.31	0.01	0.04	0.00	0.16	0.42	0.00	0.00

PG Yard	Location	Coverage	pH	NCPI	Hg	As	Cu	Ni	Zn	Pb	Cd	Cr	Pi_Hg	Pi_As	Pi_Cu	Pi_Ni	Pi_Zn	Pi_Pb	Pi_Cd	Pi_Cr
	TS184	Film	5.31	0.29	0.07	0.512	2.1	0	25	27.6	0	0	0.14	0.01	0.04	0.00	0.13	0.39	0.00	0.00
	TS185	Film	5.31	0.29	0.09	0.683	1.4	0	26.8	28.3	0	0	0.19	0.02	0.03	0.00	0.13	0.40	0.00	0.00
TS19	TS191	Open	10.4	0.75	0.83	7.29	14	31.1	37	48.9	0.8	0	0.83	0.29	0.14	0.16	0.12	0.29	1.00	0.00
	TS192	Open	10.4	1.04	1.44	4.15	11.5	33	52.8	23.5	0.3	0	1.44	0.17	0.12	0.17	0.18	0.14	0.38	0.00
	TS193	Film	9.1	0.53	0.70	5.1	23.7	32.9	40.2	69.2	0.3	0	0.70	0.20	0.24	0.17	0.13	0.41	0.38	0.00
	TS194	Open	11	0.50	0.65	2.7	16.3	33.1	77.6	49.6	0.4	0	0.65	0.11	0.16	0.17	0.26	0.29	0.50	0.00
	TS195	Film	9.7	0.56	0.26	5.28	14.2	28.4	97.8	25.3	0.6	0	0.26	0.21	0.14	0.15	0.33	0.15	0.75	0.00
TS20	TS201	Film	4.1	0.91	0.15	8.5	45.2	35.8	120.5	42.3	0.35	0	0.30	0.21	0.90	0.60	0.60	0.60	1.17	0.00
	TS202	Film	4.6	0.73	0.08	6.2	38.7	28.9	95.8	35.6	0.28	0	0.16	0.16	0.77	0.48	0.48	0.51	0.93	0.00
	TS203	Film	3.3	1.09	0.22	10.1	52.1	42.3	145.2	48.9	0.42	0	0.44	0.25	1.04	0.71	0.73	0.70	1.40	0.00
	TS204	Film	4	0.81	0.12	7.8	41.3	32.1	108.7	38.7	0.31	0	0.24	0.20	0.83	0.54	0.54	0.55	1.03	0.00
	TS205	Film	4.2	0.99	0.18	9.3	48.6	38.7	132.4	45.2	0.38	0	0.36	0.23	0.97	0.65	0.66	0.65	1.27	0.00
TS21	TS211	Open	4.1	2.88	0.13	15.6	12.6	33.4	20.2	48.6	1.2	0	0.26	0.39	0.25	0.56	0.10	0.69	4.00	0.00
	TS212	Film	5.4	0.99	0.10	2.39	24.9	37.4	24.5	44.5	0.4	0	0.21	0.06	0.50	0.62	0.12	0.64	1.33	0.00
	TS213	Open	5.8	0.58	0.08	6.95	14.2	36.8	23	45.1	0.3	0	0.17	0.17	0.28	0.53	0.12	0.50	0.75	0.00
	TS214	Open	5.8	0.75	0.11	7.64	17.5	37.7	22.7	34.6	0.4	0	0.22	0.19	0.35	0.54	0.11	0.38	1.00	0.00
	TS215	Open	4.2	1.70	0.08	31.2	11.5	35.1	22.2	26.1	0.7	0	0.17	0.78	0.23	0.59	0.11	0.37	2.33	0.00

Table S2 Raw Dataset for Vertical Distribution of Contaminants

PG Yard	Location	Depth (m)		pH	PO_4^{3-}	F^-
TS01	TS011	0～0.5	1	3.23	8.26	56.4
		5	2	3.25	10.1	746
		10	3	3.45	8.17	39.7
	TS012	0～0.5	1	2.52	45.8	708
	TS013	0～0.5	1	2.55	61.1	477
	TS014	0～0.5	1	2.67	51	179
		5	2	3	43	114
		10	3	2.8	47.7	140
	TS015	0～0.5	1	2.52	55.6	504
	TS016	0～0.5	1	2.5	43.5	43.8
	TS017	0～0.5	1	2.38	56.4	583
		5	2	2.47	59.5	340
		10	3	2.39	69.3	370
	TS018	0～0.5	1	2.98	65.6	124
TS02	TS021	4～4.5	1	5.45	1.48	5.89
		12.3～12.8	2	5.15	2.15	7.65
		20.6～21.0	3	4.65	1.29	9.73
	TS022	0.2～0.5	1	3.88	0.15	12.9
	TS023	0.2～0.5	1	4.44	0.43	21.5
TS03	TS031	0～3	1	5.3	17.1	41.8
		14～16	2	5.7	7.04	4.81
		27～30	3	5.9	3.47	4.8
	TS032	0～3	1	4.1	45.4	11
		8～10	2	3.8	3.83	7.99
		17～19	3	5.2	3.84	3.97
	TS033	0～10	1	6.2	2.67	6.64
		11～20	2	6.3	9.24	2.49
		21～30	3	4.6	122	4.38
	TS034	0～3	1	5.8	0.11	10.5
		3～10	2	6	4.47	4.32
		10～19	3	6	5.78	5.51
	TS035	0～4	1	7.3	7.91	3.7
		4～18	2	7.1	0.5	8.02
		18～30	3	7.1	4.27	4.4
TS04	TS041	0～10	1	3.9	86.7	40.2
		10～20	2	3.9	91.1	28.3
		20～30	3	3	127	77.7
	TS042	0～8	1	5.8	123	6.04
		8～16	2	6.1	43.3	7.01
		16～24	3	5.4	178	7.35
	TS043	0～6	1	5.7	51.5	2.95
		6～13	2	5.9	38.9	1.57

PG Yard	Location	Depth (m)		pH	PO_4^{3-}	F^-
		13～20	3	6	24.9	7.91
	TS044	0～6	1	6.1	5.95	4.87
		6～13	2	6.2	14.4	3.37
		13～20	3	6.4	7.47	3.47
	TS045	0.8～4	1	11	0.12	1.75
		4～8	2	11	0.07	1.66
		8～13	3	8.2	0.27	7.45
TS05	TS051	0～0.5	1	5.32	3.07	3.79
		0.5～14.5	2	5.48	3.64	5.16
		14.5～28.5	3	5.3	2.34	8.57
	TS052	0～0.5	1	6.26	2.34	2.21
		0.5～14.5	2	5.33	3.27	4.8
		14.5～28.5	3	5.94	3.7	4.16
	TS053	0～0.5	1	6	3.03	5.75
		0.5～14.5	2	5.81	2.88	11.8
		14.5～28.5	3	5.44	2.63	32
	TS054	0～0.5	1	5.92	2.85	4.09
		0.5～14.5	2	5.77	3.68	5.26
		14.5～28.5	3	5.5	5.59	3.91
	TS055	0～0.5	1	5.8	2.55	3.24
		0.5～14.5	2	5.99	3.21	4.2
		14.5～28.5	3	5.91	3.62	5.46
TS06	TS061	0.3～5.0	1	11.5	0.05	1.43
		5.0～10	2	10.4	0.44	1.08
		10～15	3	8	2.47	2.42
	TS062	0.3～3.0	1	6.6	1.63	1.15
		3.0～6.0	2	6.2	1.99	3.4
		6.0～10	3	6.3	1.35	4.68
	TS063	0～1.8	1	5.9	3.53	1.82
		1.8～3.6	2	4.4	2.06	2.17
		3.6～5.4	3	6.2	0.3	2.41
TS07	TS071	0～1.3	1	5.8	1.56	6.48
		11～12	2	6.2	2.97	3.42
		19～20	3	6.5	2.71	2.58
TS08	TS081	1.9～2.4	1	5.72	1.13	2.43
		6.3～6.8	2	5.69	0.56	1.88
		10.6～11.0	3	5.92	0.71	2.43
	TS082	0.2～0.5	1	5.59	1.04	2.14
	TS083	0.2～0.5	1	4.42	0.42	2.33
TS09	TS091	1.3～3.0	1	6.3	0.91	2.38
		3.0～11	2	6.4	0.54	6.65
		11～22	3	6.6	1.71	3.18
	TS092	1.3～3.0	1	3.1	1.08	3.51

PG Yard	Location	Depth (m)		pH	PO_4^{3-}	F^-
		3.0～20	2	4.6	0.84	2.23
		20～32	3	5.4	1.55	2.16
	TS093	0.3～10	1	6.4	1.34	12.1
		10～20	2	6.6	0.09	4.96
		20～30	3	6.8	2.7	3.68
	TS094	1.5～2.4	1	8.9	4.24	1.88
		2.4～3.0	2	8.7	2.44	2.2
		3.0～3.5	3	8.4	5.51	2.49
	TS095	0～1.2	1	8.3	3.55	2.49
		1.2～2.0	2	8.3	3.54	2.38
		3.0～3.6	3	8.2	0.78	1.26
	TS096	1.0～2.0	1	5.5	3.51	7.15
		2.0～3.0	2	4.6	4.67	10.3
		3.0～4.0	3	4.3	0.65	25.3
TS10	TS101	0～2	1	3.26	55.6	24.9
		2～6	2	3.61	43.5	21.4
		6～10	3	4.21	18.9	36.4
	TS102	0～2	1	4.81	7.65	6.71
		2～6	2	4.56	9.13	11.7
		6～10	3	3.56	2.28	142
	TS103	0～7	1	2.84	1.88	13.6
		7～14	2	3.72	3.22	41.6
		14～20	3	3.07	9.53	52.9
	TS104	0～4	1	2.60	0.48	90.2
		4～8	2	5.63	6.98	11.6
		8～12	3	4.41	10.3	11.2
	TS105	0～3	1	3.14	0.06	38.7
		3～6	2	5.01	4.83	8.57
		6+	3	4.98	4.23	3.87
TS11	TS111	0.5～7	1	5.5	49.9	3.1
		7～14	2	5.4	2.3	29
		14～20.5	3	5.8	1.34	2.51
	TS112	0.5～5.5	1	4.9	0.3	9.36
		5.5～11	2	5.5	3.38	1.62
		11～16.5	3	5.8	1.23	2.02
	TS113	1.5～5	1	5.9	1.06	1.78
		5～10	2	5.3	0.48	12.1
		10～16	3	5.3	0.34	16.3
TS12	TS121	0～2	1	3.4	10.1	104
		2～9	2	3.7	1.39	66.5
		9～17	3	3.4	7.37	74.8
	TS122	0.2～0.5	1	8	0.8	0.94
	TS123	0.2～0.5	1	3.1	22.1	135

PG Yard	Location	Depth (m)		pH	PO_4^{3-}	F^-
TS13	TS131	0～0.5	1	2.9	12	11.4
		0.5～14.5	2	3.34	12.5	27.7
		14.5～28.5	3	3.41	13.3	29.7
	TS132	0～0.5	1	3.32	81.1	4.99
		0.5～14.5	2	3.76	86.5	7.15
		14.5～28.5	3	4.16	83	14.2
	TS133	0～0.5	1	2.67	1.71	5.53
		0.5～14.5	2	4.72	2.47	4.76
		14.5～28.5	3	2.58	2.9	18.5
	TS134	0～0.5	1	2.47	12	5.62
		0.5～14.5	2	4.59	10.8	17.2
		14.5～28.5	3	4.46	12.3	10.9
	TS135	0～0.5	1	2.88	13.1	15.5
		0.5～14.5	2	2.25	14.6	44.8
		14.5～28.5	3	3.3	14.2	7.72
TS14	TS141	1.0～4.0	1	5.9	3.78	2.25
		4.0～8.0	2	5.7	1.29	2.91
		8.0～13.5	3	5.8	0.688	1.8
	TS152	0～0.5	1	5.7	0.204	1.01
	TS143	0～0.5	1	5.4	9.96	2.8
TS15	TS151	0～0.5	1	9.7	1.09	8.76
	TS152	0～0.5	1	11.9	1.39	5.9
	TS153	0～0.5	1	9.54	3.63	7.75
	TS154	0～0.5	1	9.01	2.08	13.5
	TS155	0～0.5	1	6.53	7.92	45.3
TS16	TS161	1.0～5.0	1	6.5	12	1.96
		5.0～21	2	6.5	11.5	1.71
		21～33	3	6.6	12.9	1.73
	TS162	0.5～5.0	1	5.8	6.65	4.32
		5.0～20	2	6	1.85	4.18
		20～29.5	3	6.1	0.53	1.52
	TS163	0.3～3.0	1	6.3	9.94	9.9
		3.0～32	2	6.2	14.1	0.281
		32～49	3	6.5	8.86	1.92
	TS164	0.3～16	1	6.4	18.4	2.45
		16～32	2	3.9	166	21.5
		32～48	3	5.6	34.7	0.634
	TS165	1.0～3.0	1	5.4	87.3	15.7
		3.0～12	2	5.8	22.9	1.77
		12～20	3	5.6	37.6	2
TS17	TS171	0.4～0.9	1	3.88	8.89	14.5
		1.8～2.3	2	4.45	9.09	13.4
		3.0～3.5	3	7.87	0.02	6.01

PG Yard	Location	Depth (m)		pH	PO_4^{3-}	F^-
	TS172	0.2～0.5	1	6.13	0.79	5.54
	TS173	0.2～0.5	1	6.4	0.59	3
TS18	TS181	0～0.5	1	5.89	12.4	3.51
		1.5～2	2	5.22	15.4	4.84
		2.5～3	3	3.22	337	16.1
	TS182	0～0.5	1	5.43	4.94	9.83
		4～4.5	2	4.91	8.42	14.7
		7～7.5	3	5.52	3.48	2.06
	TS183	0～0.5	1	5.81	3.69	6.57
		2.5～3	2	5.44	13.8	22.1
		5～5.5	3	5.01	19	10.8
	TS184	0～0.5	1	5.31	10.7	7.93
		3～3.5	2	5.18	28.7	7.02
		6.5～7	3	5.23	14.5	9.91
	TS185	0～0.5	1	5.31	14.3	6.44
		3～3.5	2	5.27	18.4	15.8
		6～6.5	3	5.23	15.7	13.2
TS19	TS191	0～0.3	1	10.4	0.07	1.46
	TS192	0～0.3	1	10.4	0.09	1.04
	TS193	0～0.3	1	9.1	0.12	2.26
	TS194	0～0.3	1	11	0.06	2.4
	TS195	0～0.3	1	9.7	2.39	2
TS20	TS201	0.5	1	4.1	2.86	53.6
		12	2	3.6	4.97	44.6
		24	3	3.5	2.3	34.7
	TS202	0.5	1	4.6	2.73	1.17
		9	2	4.6	1.83	2.56
		18	3	4.1	2.47	2.06
	TS203	0.5	1	3.3	1.13	25.4
		5	2	3.5	7.77	66.1
		10	3	3.2	5.88	78.6
	TS204	0.5	1	4	4.55	9.72
		15	2	4.2	4.24	6.73
		30	3	4.4	2.96	6.08
	TS205	0.5	1	4.2	16.1	14.5
		3	2	4.3	15.8	13.1
		7	3	4.1	17.8	16.4
TS21	TS211	0.5～1.5	1	4.1	12	9.11
		4～5	2	4.6	46.1	6.65
		9～10	3	4.2	99.4	8.99
	TS212	0.5～1.5	1	5.4	2.85	3.2
		3～3.5	2	5.7	7.09	2.05
		5～6	3	5.9	3.81	1.98

PG Yard	Location	Depth (m)		pH	PO_4^{3-}	F^-
	TS213	0.5～1.5	1	5.8	0.53	17
		20～21	2	5.8	33.5	5.24
		31～32	3	5.7	24	10.2
	TS214	0.5～1.5	1	5.8	0.53	7.17
		15～16	2	5.2	36.2	7.1
		31～32	3	5.4	66.3	11.7
	TS215	0.5～1.5	1	4.2	22.1	9.31
		19～20	2	2.8	130	16.5
		39～40	3	4.4	33.5	2.36

Note: The depth codes correspond as follows: 1 = Surface Layer, 2 = Middle Layer, 3 = Deep Laye

Table S3 Raw Dataset of Contaminants in Fresh PG

PG Yard	Location	pH	TP	F^-	Cu	Zn	Hg	As	Cd	Pb	Cr	Ni
TS03	G031	2.2	1180	258	0.03	0.41	0.00052	0.0282	0.00684	0.0346	0.11	0.1
	G032	2.2	1090	285	0.05	0.44	0.00072	0.0261	0.0164	0.0352	0.15	0.11
TS04	G041	6.6	67.3	22	0	0.07	0.00027	0.023	0.0011	0.0177	0	0.14
	G042	6.6	39.8	8.86	0	0	0.00026	0.014	0.00271	0.0186	0	0.13
TS10	G101	2.72	98.7	45	0.31	0.05	2.45	43.5	0.02	0.14	0.05	0.11
	G102	2.95	43.1	29.2	0.06	0.03	12.5	7.03	0.01	0.35	0.03	0.04
TS13	G131	2.49	130	58	0.09	0.32	0.00141	0.0397	0.02	0.15	0.07	0.1
	G132	2.43	123	65.1	0.1	0.36	0.00023	0.0284	0.02	0.15	0.08	0.11
TS15	G151	3.9	53.1	132	0.13	0.12	0.00008	0.0186	0	0	0	0
	G152	3.17	53.3	186	0.14	0.18	0.00005	0.0178	0	0.04	0	0
TS16	G161	5.5	16.3	0	0	0.087	0.00098	0.00676	0.00107	0.0264	0.03	0
	G162	3.8	29.2	0	0.07	0.253	0.00114	0.00916	0.00194	0.0293	0.03	0.05
TS20	G201	7.5	10.1	9.11	0	0	0.00012	0.52	0	0	0	0
	G202	7.6	9.89	3.5	0	0	0.00015	0.55	0	0	0	0
	G203	7.7	10.3	5.74	0.005	0	0.00018	0.98	0	0	0	0.12
	G204	7.5	9.73	1.8	0	0	0.00024	0.54	0	0	0	0
	G205	7.4	10.9	9.45	0	0	0.00021	0.61	0	0	0	0.1
TS21	G211	3.9	74.8	14.8	0.02	0.42	0.00082	0.0166	0.00076	0.0274	0	0
	G212	3.5	85.5	13.5	0.02	0.21	0.00053	0.0214	0.00105	0.03	0	0.04

Table S4 Raw Data on Pollutant Constituents in PG-L

PG Yard	pH	TP	F^-	Cu	Zn	Hg	As	Cd	Pb	Cr	Ni
TS03	1.8	43.5	488	4.38	48.6	0.00107	0.0093	0.395	0.221	0.423	9.78
TS04	3.8	111	133	0.576	0.865	0.00056	0.0005	0.083	0	0.113	2.22
TS10	2.6	390	410	0.407	5.85	0.00087	1.37	0.488	0.00386	0.399	22.6
TS13	2.1	4630	482	1.22	15.6	0.154	0.622	0.647	0.0856	1.25	7.8
TS15	2.4	1540	722	0.2	5.92	0.135	0.421	0.0911	0.152	0.43	1.16
TS16	6.3	68.2	36.6	0.13	0.983	0.0022	0.261	0.062	0	0	1.1
TS20	2.7	11.7	156	0.032	0.89	0	13.9	0	0.28	0.028	0.81
TS21	5.3	344	24.3	0	0	0.00065	0.0788	0	0	0	0.235

Table S5 Data Quality Control (QC)

Analyte	Method Code	Detection Limit	Unit	Detection Limit	Unit
TP	GB 11893-89	/	/	0.01	mg/L
Cd	HJ 781-2016	0.1	mg/kg	0.01	mg/L
Cr	HJ 781-2016	0.5	mg/kg	0.02	mg/L
Cu	HJ 781-2016	0.4	mg/kg	0.01	mg/L
Ni	HJ 781-2016	0.4	mg/kg	0.02	mg/L
Pb	HJ 781-2016	1.4	mg/kg	0.03	mg/L
Hg	HJ 702-2014	0.002	μg/g	0.02	μg/L
As	HJ 702-2014	0.010	μg/g	0.10	μg/L
Zn	HJ 786-2016	2.0	mg/kg	0.06	mg/L
